# Incremental Diagnostic Yield of Fetal MRI After Targeted Neurosonography in Fetuses with Agenesis of the Corpus Callosum: A Retrospective Cohort Study

**DOI:** 10.3390/jcm15218502

**Published:** 2026-11-02

**Authors:** Serdar Aykut, İsmail Cüneyt Evrüke, Süleyman Cansun Demir, Aslıhan Kurt, Ahmet Zeki Nessar, Ayşe Büşra Önder

**Affiliations:** 1 Division of Perinatology, Department of Obstetrics and Gynecology, Mardin Training and Research Hospital, Mardin 47100, Türkiye; 2 Division of Perinatology, Department of Obstetrics and Gynecology, Çukurova University Faculty of Medicine, Adana 01330, Türkiye; cuneytevruke@gmail.com (İ.C.E.); cansundemir@gmail.com (S.C.D.); 3 Division of Perinatology, University of Health Sciences, Gaziantep City Hospital, Gaziantep 27060, Türkiye; 4 Division of Perinatology, Department of Obstetrics and Gynecology, Osmaniye State Hospital, Osmaniye 80000, Türkiye; 5 Division of Gynecologic Oncology, Department of Obstetrics and Gynecology, Mardin Training and Research Hospital, Mardin 47100, Türkiye; aysebusraonder@hotmail.com

**Keywords:** agenesis of the corpus callosum, fetal magnetic resonance imaging, neurosonography, prenatal diagnosis, ventriculomegaly

## Abstract

**Background/Objectives**: This study aimed to assess additional findings reported on fetal magnetic resonance imaging (MRI) after targeted neurosonography in fetuses with agenesis of the corpus callosum (ACC). **Methods**: This retrospective cohort included 129 singleton pregnancies with complete or partial ACC evaluated at a tertiary referral center between January 2020 and January 2023. All underwent fetal MRI within 7 days of neurosonography. Incremental findings were abnormalities not identified on the preceding examination. Final isolated or non-isolated classification incorporated both modalities. **Results**: MRI reported at least one incremental intracranial or craniofacial finding in 42/129 fetuses (32.6%). The most frequent findings were cerebellar hypoplasia (15/129, 11.6%), vermian agenesis (11/129, 8.5%), and arachnoid cysts (9/129, 7.0%). Final classification identified 71 isolated and 58 non-isolated cases. Among 77 tested fetuses, 15 had aneuploidy; the frequency was higher in non-isolated cases (14/37 versus 1/40; *p* < 0.001). **Conclusions**: MRI provided additional anatomical information after neurosonography in approximately one-third of this referral cohort. These findings support assessment beyond confirmation of ACC, but do not establish diagnostic accuracy, improved parental decisions, or long-term prognostic benefit.

## 1. Introduction

Agenesis of the corpus callosum (ACC) is a heterogeneous congenital brain abnormality that may occur as an isolated finding or in association with other central nervous system, extracranial, and genetic abnormalities. This distinction is clinically important because the presence and nature of associated abnormalities are major determinants of prognosis and prenatal counseling. Although truly isolated ACC is generally associated with a more favorable outcome, normal neurodevelopment cannot be guaranteed, and subtle cognitive, language, behavioral, or social difficulties may become apparent later in childhood [1].

Targeted neurosonography is the primary imaging modality for the prenatal evaluation of suspected ACC and enables detailed assessment of callosal morphology, the ventricular system, midline structures, and associated fetal abnormalities. Nevertheless, some abnormalities involving cortical development, neuronal migration, the posterior fossa, or other intracranial structures may remain undetected on ultrasonography. Current guidelines therefore recommend fetal magnetic resonance imaging (MRI) as a complementary examination when a fetal cerebral abnormality is suspected and when additional anatomical information may influence diagnosis, prognosis, or parental counseling [2].

The additional diagnostic contribution of fetal MRI following expert neurosonography remains clinically relevant but varies according to patient selection, imaging protocols, operator expertise, gestational age, and the definition of an additional finding. A systematic review and meta-analysis demonstrated that fetal MRI can identify previously unrecognized intracranial abnormalities in fetuses considered to have isolated ACC on prenatal ultrasonography [3]. Similarly, a multinational study by the ENSO Working Group reported that fetal MRI detected additional central nervous system abnormalities in 11.2% of fetuses with apparently isolated callosal anomalies following expert neurosonography, with malformations of cortical development representing the most frequent additional findings [4]. Genetic evaluation is also essential because pathogenic variants may be identified even in apparently isolated cases. A recent systematic review and meta-analysis reported an incremental diagnostic yield of 43% for prenatal exome sequencing after a negative chromosomal microarray result in fetuses with ACC [5].

However, relatively few studies have evaluated the incremental diagnostic yield of fetal MRI in a cohort in which all fetuses with complete or partial ACC underwent both detailed neurosonography and fetal MRI within a standardized tertiary referral setting. Therefore, this study aimed to determine the incremental diagnostic yield of fetal MRI after detailed ultrasonography and targeted neurosonography in fetuses with prenatally diagnosed ACC. Secondary objectives were to describe associated ultrasound and MRI findings and genetic results according to the final imaging phenotype.

## 2. Materials and Methods

### 2.1. Study Design and Setting

This retrospective cohort study was conducted at the Division of Perinatology, Department of Obstetrics and Gynecology, Çukurova University Faculty of Medicine, a tertiary referral center located in southern Türkiye. The center provides advanced prenatal diagnostic and management services for pregnancies referred from several surrounding provinces because of suspected or confirmed fetal abnormalities. The study is reported in accordance with the Strengthening the Reporting of Observational Studies in Epidemiology (STROBE) statement.

The study included pregnancies evaluated between 1 January 2020 and 6 January 2023. Following ethics committee approval, clinical, ultrasonographic, fetal MRI, genetic, pregnancy outcome, and delivery data from eligible cases were retrospectively retrieved from institutional medical records and analyzed in a de-identified manner. Approximately 5800 individual pregnancies were evaluated for prenatal diagnosis at the center during this same period, excluding repeat visits. This approximate denominator was used only to describe the frequency of ACC in the referral population, not to estimate population-based incidence.

### 2.2. Study Population

The study population comprised 129 singleton pregnancies with complete or partial agenesis of the corpus callosum confirmed by detailed fetal structural examination and targeted neurosonography at our center. Eligible cases included pregnancies referred from surrounding provinces with suspected or established agenesis of the corpus callosum and pregnancies under follow-up at our institution in which the abnormality was detected during prenatal ultrasonography. ACC was confirmed in all referred cases, and all 129 fetuses underwent fetal cranial magnetic resonance imaging; no eligible case was excluded from the analysis. All eligible pregnancies meeting these criteria during the prespecified study period were included; therefore, no a priori sample-size calculation was performed.

### 2.3. Prenatal Ultrasonography and Targeted Neurosonography

Because our institution is a tertiary referral center for fetal abnormalities, all cases were jointly evaluated by a maternal–fetal medicine specialist and a maternal–fetal medicine fellow. Each fetus underwent a detailed structural examination and targeted neurosonography using a Voluson 730 Pro ultrasound system (GE Healthcare, Zipf, Austria) equipped with a 4–8-MHz convex transducer.

The diagnosis of complete or partial agenesis of the corpus callosum was based on direct and indirect sonographic findings obtained in the appropriate imaging planes. The fetal brain was systematically assessed for associated intracranial abnormalities. The examination also included a detailed evaluation of fetal anatomy to identify major extracranial structural abnormalities and minor sonographic markers. Ventricular width was measured at the level of the atrium of the lateral ventricle. A major structural abnormality was defined as a structural defect expected to influence fetal prognosis or pregnancy management or to require postnatal medical or surgical evaluation. Minor anomalies and soft markers were defined as sonographic findings that, when isolated, were not considered major structural abnormalities.

### 2.4. Fetal MRI

All fetuses with a confirmed sonographic diagnosis of complete or partial agenesis of the corpus callosum underwent fetal cranial MRI, irrespective of the presence or absence of additional abnormalities on ultrasonography. Fetal MRI was performed within 7 days after the detailed ultrasound examination and targeted neurosonography conducted at our center. Examinations were performed using a 3.0-T MRI system (Ingenia Elition, Philips Healthcare, Best, The Netherlands) according to the institution’s standard clinical fetal brain imaging protocol. Initial localization images were obtained in three orthogonal planes relative to the maternal imaging axes using wide-field-of-view T2-weighted single-shot turbo spin-echo sequences, with a field of view of 320–400 mm, a section thickness of 6–8 mm, and an interslice gap of 1–2 mm. These images were used to determine fetal position and plan the subsequent fetal brain acquisitions. The fetal region of interest was positioned as close as possible to the center of the receiver coil to optimize signal intensity, and maternal or coil positioning was adjusted when necessary.

Motion-robust diagnostic T2-weighted single-shot turbo spin-echo acquisitions were subsequently obtained in axial, coronal, and sagittal planes relative to the fetal brain and constituted the principal sequences for anatomical assessment. Diagnostic T2-weighted images were acquired with a section thickness of 3–4 mm and an interslice gap of 0–0.5 mm, with approximately 20–25 slices obtained in each imaging plane. T1-weighted sequences were routinely acquired as complementary sequences. Imaging planes and acquisition parameters were adapted to gestational age, fetal position, and the region of interest, and sequences affected by fetal motion were repeated when necessary. The total examination time was approximately 30–40 min. Neither maternal nor fetal sedation was administered. All examinations were interpreted by the same radiologist experienced in fetal neuroimaging.

Fetal MRI findings were extracted from the original clinical radiology reports issued at the time of examination; the complete imaging dataset was not retrospectively reinterpreted for this study. All reported findings were interpreted in relation to gestational age at MRI. Diagnostic morphological principles established in fetal neurosonography were considered only when the corresponding anatomical landmarks could be reliably visualized on multiplanar fetal MRI. In routine reporting, cerebellar hypoplasia was determined using a combined quantitative and qualitative assessment of gestational-age-adjusted cerebellar dimensions, overall size, symmetry, contour, and tissue architecture, with reference to published fetal MRI normative data [6]. Vermian abnormalities were assessed on midsagittal and orthogonal images according to gestational-age-appropriate size and morphology using modality-specific prenatal imaging reference data [7]. Intracranial cysts were characterized according to their anatomical location, morphology, signal characteristics, relationship to adjacent structures, and associated mass effect. Cerebral volume loss was reported on the basis of qualitative morphological features, supported by available gestational-age-adjusted quantitative measurements when documented in the clinical examination.

For the purposes of this study, an MRI finding was classified as an incremental intracranial or craniofacial finding only when it had not been identified during the preceding detailed ultrasound examination and targeted neurosonography performed at our center. Findings already demonstrated by ultrasonography were not classified as incremental MRI findings.

### 2.5. Imaging-Based Classification

Cases were classified as isolated or non-isolated according to the combined findings of ultrasonography and fetal MRI. Isolated agenesis of the corpus callosum was defined as the absence of an additional intracranial or craniofacial abnormality on fetal MRI and the absence of a major extracranial structural abnormality on ultrasonography.

Non-isolated agenesis of the corpus callosum was defined by the presence of at least one additional intracranial or craniofacial abnormality detected on fetal MRI or a major extracranial structural abnormality identified on ultrasonography. Ventriculomegaly and colpocephaly were regarded as findings within the phenotypic spectrum of agenesis of the corpus callosum and, when present alone, did not result in classification as non-isolated. Minor sonographic markers were recorded separately and were not, in isolation, considered sufficient for classification as non-isolated.

### 2.6. Genetic Evaluation

Invasive prenatal diagnostic testing was offered following multidisciplinary counseling. In pregnancies in which diagnostic testing was accepted, amniocentesis was performed at or before 24 weeks of gestation, whereas cordocentesis was performed after 24 weeks of gestation, in accordance with the institutional clinical protocol. Conventional karyotyping and chromosomal microarray analysis were performed concurrently on all obtained fetal samples. Genetic results were categorized as normal karyotype and chromosomal microarray results, variant of uncertain significance, or aneuploidy. Pregnancies without an available genetic test result were excluded from analyses requiring genetic information. Genetic findings were categorized according to the contemporaneous clinical laboratory reports; no study-specific retrospective reinterpretation or reclassification of variants was performed.

### 2.7. Clinical Counseling

Following the sonographic diagnosis, fetal MRI was used to confirm ACC and assess associated abnormalities. Termination decisions were made after review of the MRI findings and counseling of the family regarding possible adverse outcomes and prognostic uncertainty. Decisions were not deferred pending fetal karyotype or chromosomal microarray results. This clinical sequence is reported for context; the study did not evaluate the effect of MRI on parental decisions.

### 2.8. Statistical Analysis

Continuous variables were summarized as median and interquartile range (IQR), and categorical variables as *n/N* (%) or n (%), with denominators stated in the table headings or footnotes. Continuous variables were compared using the Mann–Whitney U test. Two-by-two categorical comparisons used Pearson’s chi-square test without continuity correction when all expected cell frequencies were at least 5; otherwise, a two-sided Fisher’s exact test was used. The test applied to each comparison is identified in the tables. Each pair of complementary categories is represented by a single *p* value. Analyses used available-case data without imputation. All tests were two-sided, and *p* < 0.05 was considered statistically significant. Pregnancy and delivery outcomes were not analyzed as study endpoints.

### 2.9. Ethical Approval

The study was conducted in accordance with the Declaration of Helsinki and was approved by the Çukurova University Faculty of Medicine Non-Interventional Clinical Research Ethics Committee (Meeting No. 129; Decision No. 33; 6 January 2023). The requirement for study-specific informed consent was waived by the ethics committee because of the retrospective design and the use of de-identified clinical data.

## 3. Results

During the study period, approximately 5800 individual pregnancies were evaluated for prenatal diagnosis at our center. The 129 pregnancies with ACC represented approximately 2.2% of this referral population. This proportion should not be interpreted as a population-based incidence estimate. A total of 129 pregnancies met the eligibility criteria and were included; no eligible case was excluded. Based on the combined ultrasonographic and fetal MRI findings, 71 cases (55.0%) were classified as isolated ACC and 58 (45.0%) as non-isolated ACC. The median maternal age was 28.0 years (interquartile range [IQR], 24.0–33.0), and the median gestational age at diagnosis was 24.0 weeks (IQR, 21.0–30.0). Forty-four women (34.1%) were nulliparous and 85 (65.9%) were multiparous. Complete ACC was identified in 116 fetuses (89.9%), whereas 13 (10.1%) had partial ACC. Ventriculomegaly was present in 98 cases (76.0%), with a median ventricular atrial width of 12.0 mm (IQR, 9.0–14.0). Maternal age, gestational age at diagnosis, parity, ACC type, ventriculomegaly, ventricular atrial width, and the frequency of minor anomalies or soft markers did not differ significantly between the isolated and non-isolated groups (all *p* > 0.05) (Table 1).

Fetal MRI reported at least one incremental intracranial or craniofacial finding in 42/129 fetuses (32.6%). The most frequent findings were cerebellar hypoplasia (15/129, 11.6%), vermian agenesis (11/129, 8.5%), and arachnoid cysts (9/129, 7.0%). Individual fetuses could have more than one finding. Ultrasound identified at least one additional major structural abnormality in 29/129 fetuses (22.5%) (Table 2). The two panels are descriptive and were not directly compared because the MRI panel contains incremental intracranial or craniofacial findings, whereas the ultrasound panel contains additional major structural abnormalities in different anatomical domains.

Invasive prenatal diagnostic testing was performed in 77 of 129 fetuses (59.7%). Amniocentesis was performed in 61 of the 77 tested pregnancies (79.2%), whereas cordocentesis was performed in 16 (20.8%). The proportion of fetuses undergoing invasive testing did not differ significantly between the final isolated and non-isolated ACC groups (56.3% vs. 63.8%, respectively; *p* = 0.391). Among fetuses with an available genetic result, both conventional karyotyping and chromosomal microarray analysis were normal in 54 cases (70.1%), a variant of uncertain significance was identified in 8 (10.4%), and aneuploidy was detected in 15 (19.5%). Aneuploidy was significantly more frequent in the non-isolated group than in the isolated group (37.8% vs. 2.5%; *p* < 0.001). Trisomy 18 was identified exclusively in fetuses with non-isolated ACC (21.6% vs. 0%; *p* = 0.002), whereas the frequencies of trisomy 13 and trisomy 21 did not differ significantly between the groups. Variants of uncertain significance were detected in 12.5% of isolated cases and 8.1% of non-isolated cases (*p* = 0.713). Genetic testing was declined in 52 pregnancies (40.3%) (Table 3).

## 4. Discussion

The principal finding was that fetal MRI reported at least one incremental intracranial or craniofacial abnormality in 42/129 fetuses (32.6%) after targeted neurosonography. Posterior fossa abnormalities, particularly cerebellar hypoplasia and vermian agenesis, and intracranial cysts were the most frequent findings. The final imaging phenotype was non-isolated in 58/129 cases (45.0%). These results describe the additional anatomical information reported by MRI in a tertiary referral cohort; without standardized postnatal confirmation, they do not establish sensitivity, specificity, or diagnostic accuracy.

Fetal MRI is recommended as a complementary modality when a fetal cerebral abnormality is suspected on ultrasonography and additional anatomical characterization may influence diagnosis and counseling [2]. A systematic review and meta-analysis showed that fetal MRI can reveal additional intracranial abnormalities in fetuses considered to have isolated ACC following prenatal ultrasonography [3]. The ENSO study identified additional CNS abnormalities on fetal MRI in 11.2% (30/269) of fetuses with apparently isolated ACC after expert multiplanar neurosonography and a normal karyotype; both complete and partial ACC were included [4]. Our cohort included isolated and non-isolated presentations and did not require a normal karyotype. Moreover, our endpoint included craniofacial findings in addition to intracranial abnormalities. The overall rate of 32.6% in our cohort and the ENSO estimate are therefore descriptive results from different case mixes and endpoint definitions, rather than evidence of superior diagnostic performance. An ENSO-comparable subgroup defined before MRI is not reported in the present analysis; the final isolated group, classified using both modalities, cannot serve as a substitute. Differences in genetic selection, gestational age, and ultrasound protocols further limit direct comparison.

The predominance of posterior fossa abnormalities and intracranial cysts in our cohort emphasizes that fetal MRI assessment should extend beyond confirmation of callosal agenesis. Cerebellar hypoplasia and vermian abnormalities may be difficult to characterize completely with ultrasonography because of fetal position, acoustic shadowing, gestational age, and limitations in visualization of the posterior fossa. MRI provides multiplanar visualization and improved tissue contrast without acoustic-window limitations. Nevertheless, MRI and targeted neurosonography should be regarded as complementary rather than competing examinations. The diagnostic contribution of MRI depends substantially on the quality of the preceding neurosonographic examination and the expertise of both the fetal medicine and radiology teams.

Additional anatomical information obtained from MRI may also improve prognostic stratification. Diogo et al. developed a fetal MRI-based anatomical scoring system incorporating abnormalities beyond the corpus callosum and demonstrated associations between the fetal MRI phenotype and subsequent cognitive, language, and motor outcomes [8]. Shwe et al. similarly showed that the distinction between isolated and complex ACC on fetal and postnatal MRI was associated with clinical and neurodevelopmental outcomes [9]. Although our study did not include standardized postnatal neuroimaging or long-term neurodevelopmental assessment, the identification of previously unrecognized intracranial findings provides additional information for anatomical classification. This study did not assess whether MRI changed counseling, parental decisions, or clinical management. However, our data do not establish that every incremental MRI finding independently alters prognosis or pregnancy management.

Genetic abnormalities represent another important component of the evaluation of fetal ACC. In our cohort, genetic testing was performed in 77 of 129 pregnancies, and aneuploidy was identified in 15 of the 77 tested fetuses. Aneuploidy was markedly more frequent among non-isolated cases than among isolated cases (37.8% vs. 2.5%). This finding supports the close relationship between additional structural abnormalities and an abnormal chromosomal result. However, the low aneuploidy rate among apparently isolated cases should not be interpreted as the absence of genetic risk. Heide et al. identified pathogenic single-nucleotide variants and copy-number variants in fetuses with callosal abnormalities, demonstrating that conventional karyotyping alone cannot identify the full spectrum of genetic etiologies [10]. Prenatal exome sequencing may provide additional diagnoses in fetuses with central nervous system abnormalities after nondiagnostic karyotype and chromosomal microarray results [11]. A recent systematic review and meta-analysis reported an incremental exome-sequencing yield of 43% after negative chromosomal microarray analysis, with yields of 55% in ACC associated with extracranial abnormalities, 43% in ACC with additional cranial abnormalities, and 32% in apparently isolated ACC [5]. Contemporary fetal cohorts have likewise demonstrated marked genetic heterogeneity and a higher diagnostic yield in non-isolated cases [12]. Therefore, parental counseling should address the limitations of conventional cytogenetic testing and the potential role of chromosomal microarray analysis and exome sequencing, particularly when additional abnormalities are present.

### Clinical Implications

In clinical practice, MRI should complement expert neurosonography by addressing the remaining anatomical questions in an individual fetus with ACC, consistent with ISUOG guidance [2]. Our findings highlight the importance of reviewing the posterior fossa and vermis, intracranial cysts, and cortical development rather than limiting MRI to confirmation of callosal absence. Cerebellar and vermian findings should be interpreted using gestational-age-appropriate size and morphology, with published MRI reference data where applicable [6,7]. These are considerations for clinical assessment, not new validated MRI referral criteria derived from this cohort. All included fetuses underwent MRI, so the study cannot determine which subgroup can safely omit the examination.

For prenatal counseling, the combined imaging findings can be used to describe whether ACC appears anatomically isolated or is accompanied by other findings. The potential significance and uncertainty of each associated finding should be discussed individually. MRI phenotype has been associated with later outcomes in previous studies [8,9], but our study did not validate lesion-specific prognostic estimates or measure a change in counseling or parental decisions. Additional findings should not automatically be interpreted as evidence of a poor outcome or as an indication for termination. Conversely, apparently isolated ACC does not guarantee normal neurodevelopment, and counseling should incorporate genetic findings and the limitations of prenatal imaging [1].

Prenatal counseling remains particularly challenging when ACC appears isolated. A systematic review reported normal neurodevelopment in most children with prenatally diagnosed isolated ACC, although a clinically important minority subsequently developed motor, language, cognitive, behavioral, or epilepsy-related difficulties [13]. Long-term follow-up studies have shown that subtle impairments in executive functioning, language, memory, academic performance, and social cognition may become evident only at school age or adolescence [14,15]. Contemporary counseling recommendations therefore emphasize that apparently isolated ACC is associated with a more favorable prognosis than non-isolated ACC but does not guarantee normal neurodevelopment [1]. Similarly, complete and partial ACC should not be assumed to have uniformly different outcomes based solely on callosal morphology. A recent study of partial ACC demonstrated heterogeneous neurological outcomes and indicated that associated structural and genetic abnormalities may be more informative than the extent of callosal absence alone [16].

The strengths of this study include its relatively large single-center cohort, the inclusion of both complete and partial ACC, fetal MRI evaluation of all included fetuses, and the short interval between targeted neurosonography and MRI. All cases underwent repeat detailed assessment at a tertiary referral center by a maternal–fetal medicine specialist and a maternal–fetal medicine fellow, and all MRI examinations were interpreted by the same radiologist with specific experience in fetal central nervous system abnormalities. The combined assessment of ultrasound findings, incremental MRI findings, and genetic results provides a clinically relevant description of prenatally diagnosed ACC.

Several limitations should be acknowledged. The retrospective, single-center referral design introduces selection and information bias and limits generalizability. The approximate frequency of ACC among pregnancies evaluated at our center reflects a selected referral population and cannot establish regional or population-based incidence. MRI findings were extracted from contemporaneous clinical reports; ultrasound and MRI datasets were not independently reassessed in a blinded research setting. Neither standardized postnatal neuroimaging nor long-term neurodevelopmental follow-up was available, so reported incremental findings could not be systematically validated and their prognostic significance remains uncertain. Interpretation by one experienced radiologist improved consistency but precluded assessment of interobserver agreement. Invasive genetic testing was declined in 52 fetuses, and exome sequencing was not routinely available; an anatomically isolated phenotype therefore does not exclude a genetic disorder. Comparison with ENSO remains descriptive because a subgroup with harmonized pre-MRI classification, genetic eligibility, and CNS-only endpoints is not presented. MRI was reviewed before termination decisions, but the study did not measure its effect on decision-making. Nonsignificant comparisons do not establish equivalence.

## 5. Conclusions

Fetal MRI reported additional intracranial or craniofacial findings in 42/129 fetuses (32.6%) after targeted neurosonography in this tertiary referral cohort. Posterior fossa abnormalities and intracranial cysts were the most frequent incremental findings. These results support a comprehensive MRI assessment beyond confirmation of ACC and integration of imaging and genetic information in prenatal counseling. The reported rate reflects this cohort and its endpoint definition and should not be interpreted as superior diagnostic performance compared with differently selected cohorts. Standardized postnatal confirmation and long-term follow-up are needed to establish the accuracy and prognostic significance of individual MRI findings.

## Figures and Tables

**Table 1:** Maternal, obstetric, and sonographic characteristics according to ACC classification.

Characteristic	Overall (N = 129)	Isolated ACC (n = 71)	Non-Isolated ACC (n = 58)	*p* Value
Maternal age, years	28.0 (24.0–33.0)	28.0 (24.0–34.0)	27.0 (24.0–32.0)	0.687 ^b^
Gestational age at diagnosis, weeks	24.0 (21.0–30.0)	24.0 (22.0–30.0)	24.0 (21.0–29.0)	0.652 ^b^
**Parity**				0.506 ^a^
Nulliparous	44 (34.1)	26 (36.6)	18 (31.0)	
Multiparous	85 (65.9)	45 (63.4)	40 (69.0)	
**ACC type**				0.278 ^a^
Complete ACC	116 (89.9)	62 (87.3)	54 (93.1)	
Partial ACC	13 (10.1)	9 (12.7)	4 (6.9)	
Ventriculomegaly	98 (76.0)	57 (80.3)	41 (70.7)	0.205 ^a^
Ventricular atrial width, mm	12.0 (9.0–14.0)	12.0 (10.0–14.0)	11.0 (8.0–13.0)	0.192 ^b^
Minor anomaly or soft marker	31 (24.0)	14 (19.7)	17 (29.3)	0.205 ^a^

Data are presented as median (interquartile range) or number (%). Final isolated ACC was defined on the basis of combined ultrasonographic and fetal MRI findings as ACC without an additional intracranial or craniofacial abnormality on fetal MRI or a major extracranial structural abnormality on prenatal ultrasonography. Colpocephaly and ventriculomegaly accompanying ACC were not considered additional anomalies for this classification. All tests were two-sided; *p* < 0.05 was considered statistically significant. Superscripts identify the test used: ^a^, Pearson’s chi-square test without continuity correction; ^b^, Mann–Whitney U test. A single *p* value is reported for complementary categories. Nonsignificant results do not establish equivalence. Bold text identifies category headings.

**Table 2:** Incremental Intracranial and Craniofacial Findings Detected by Fetal MRI and Additional Major Structural Abnormalities Identified by Prenatal Ultrasonography.

(**A**) Incremental intracranial and craniofacial findings detected by fetal MRI	
**Imaging Finding**	**n (%)**
At least one incremental intracranial or craniofacial MRI finding	**42 (32.6)**
**Posterior fossa abnormalities**	
Cerebellar hypoplasia	**15 (11.6)**
Vermian agenesis	**11 (8.5)**
**Intracranial cysts**	
Arachnoid cyst	**9 (7.0)**
Interhemispheric cyst	**4 (3.1)**
**Other intracranial or craniofacial findings**	
Trigonocephaly	**1 (0.8)**
Nodular heterotopia	**1 (0.8)**
Cerebral atrophy	**1 (0.8)**
Unilateral ventricular hypoplasia	**1 (0.8)**
Hypotelorism	**1 (0.8)**
(**B**) Additional major structural abnormalities identified by prenatal ultrasonography	
**At least one additional major structural finding**	**29 (22.5)**
**Central nervous system and craniofacial abnormalities**	
Microcephaly	**9 (7.0)**
Microphthalmia	**1 (0.8)**
**Cardiothoracic abnormalities**	
Congenital heart disease	**14 (10.9)**
Diaphragmatic hernia	**1 (0.8)**
**Gastrointestinal and abdominal abnormalities**	
Esophageal or gastrointestinal atresia	**2 (1.6)**
Abdominal wall anomaly	**1 (0.8)**
Enteric duplication cyst	**1 (0.8)**
**Other structural abnormalities**	
Skeletal-system anomaly	**1 (0.8)**
Genitourinary-system anomaly	**1 (0.8)**
Situs inversus	**1 (0.8)**

Data are presented as number (percentage) and percentages were calculated using the entire cohort (N = 129) as the denominator. Incremental MRI findings were defined as intracranial or craniofacial abnormalities detected on fetal MRI that had not been identified during the preceding detailed ultrasonographic examination and targeted neurosonography performed at our center. Individual fetuses could have more than one MRI or ultrasonographic finding; therefore, the sum of individual abnormalities may exceed the number of fetuses with at least one finding. Ventriculomegaly and colpocephaly were considered part of the ACC phenotype and were not included as additional abnormalities in this table. MRI and ultrasonography findings are reported descriptively; no direct statistical comparison was made because the two panels concern different anatomical findings.

**Table 3:** Invasive genetic testing and genetic results according to the final imaging phenotype.

Genetic Variable	Overall	Isolated ACC	Non-Isolated ACC	*p* Value
Invasive genetic testing performed	77/129 (59.7)	40/71 (56.3)	37/58 (63.8)	0.391 ^a^
Genetic testing declined	52/129 (40.3)	31/71 (43.7)	21/58 (36.2)	—
**Genetic results among tested fetuses**	**n = 77**	**n = 40**	**n = 37**	
Normal karyotype and chromosomal microarray results	54/77 (70.1)	34/40 (85.0)	20/37 (54.1)	0.003 ^a^
Variant of uncertain significance	8/77 (10.4)	5/40 (12.5)	3/37 (8.1)	0.713 ^b^
Any aneuploidy	15/77 (19.5)	1/40 (2.5)	14/37 (37.8)	<0.001 ^a^
Trisomy 13	3/77 (3.9)	0/40 (0.0)	3/37 (8.1)	0.106 ^b^
Trisomy 18	8/77 (10.4)	0/40 (0.0)	8/37 (21.6)	0.002 ^b^
Trisomy 21	4/77 (5.2)	1/40 (2.5)	3/37 (8.1)	0.346 ^b^

Data are presented as n/N (%) or n (%). Percentages for invasive genetic testing and declined testing were calculated using the total cohort or the corresponding imaging group as the denominator. Percentages for normal genetic results, variants of uncertain significance, aneuploidy, and individual trisomies were calculated only among fetuses with an available genetic result (overall, n = 77; isolated ACC, n = 40; non-isolated ACC, n = 37). Normal results, variants of uncertain significance, and aneuploidy were mutually exclusive categories. All tests were two-sided. ACC, agenesis of the corpus callosum; VUS, variant of uncertain significance. Superscripts identify the test used: ^a^, Pearson’s chi-square test without continuity correction; ^b^, Fisher’s exact test. A single *p* value is reported for complementary categories. Nonsignificant results do not establish equivalence.

## Data Availability

The data that support the findings of this study are available from the corresponding author upon reasonable request.
